# Vocal fold oscillation pattern changes related to loudness in patients with vocal fold mass lesions

**DOI:** 10.1186/s40463-020-00481-y

**Published:** 2020-11-23

**Authors:** Matthias Echternach, Michael Döllinger, Marie Köberlein, Liudmila Kuranova, Donata Gellrich, Marie-Anne Kainz

**Affiliations:** 1 Division of Phoniatrics and Pediatric Audiology, Department of Otorhinolaryngology Head & Neck Surgery, Munich University Hospital (LMU), Marchioninistr. 15, 81377 Munich, Germany; 2 Division of Phoniatrics and Pediatric Audiology at the Department of Otorhinolaryngology Head & Neck Surgery, University Hospital Erlangen, Medical School, Bohlenplatz 21, 91054 Erlangen, Germany; 3 Institute of Musicians’ Medicine, Freiburg University Hospital and Faculty of Medicine Freiburg University, Elsässerstr 2m, Freiburg, Germany

**Keywords:** Vocal fold mass lesions, Loudness, High-speed imaging, EGG

## Abstract

**Introduction:**

Vocal fold mass lesions can affect vocal fold oscillation patterns and therefore voice production. It has been previously observed that perturbation values from audio signals were lower with increased loudness. However, how much the oscillation patterns change with gradual alteration of loudness is not yet fully understood.

**Material and methods:**

Eight patients with vocal fold mass lesions were asked to perform a glide from minimum to maximum loudness on the vowel /i/, *ƒ*_o_ of 125 Hz for male or 250 Hz for female voices. During phonation the subjects were simultaneously recorded with transnasal high speed videoendoscopy (HSV, 20,000 fps), electroglottography (EGG), and an audio recording. Based on the HSV material the Glottal Area Waveform (GAW) was segmented and GAW parameters were computed.

**Results:**

The greatest vocal fold irregularities were observed at different values between minimum and maximum sound pressure level. There was a relevant discrepancy between the HSV and EGG derived open quotients. Furthermore, the EGG derived sample entropy and GAW values also evidenced different behavior.

**Conclusions:**

The amount of vocal fold irregularity changes with varying loudness. Therefore, any evaluation of the voice should be performed under different loudness conditions. The discrepancy between EGG and GAW values appears to be much stronger in patients with vocal fold mass lesions than those with normal physiological conditions.

**Level of evidence:**

4.

## Introduction

Vocal fold mass lesions are a main cause of dysphonia [1] and as such many histopathological findings such as polyps, nodes, cysts or oedemas frequently need medical therapy [1]. In some cases, traditional treatment such as pharmacotherapeutical approaches or voice therapy might be considered helpful. For others, however, phonomicrosurgery is often recommended [1].

Vocal fold mass lesions might induce changes to vocal fold stiffness and mass, which alter the oscillatory *eigenmode* and spatiotemporal regularity [2]. The consequent entrainment of both vocal fold oscillation patterns, which is influenced mainly by vertical vocal fold deflection [3], might be impaired, resulting in a disturbed structure of glottal air pulse generation. Furthermore, asymmetries might arise which influence the strength of the intraglottal vortices and, in turn, vocal efficiency [4]. In addition, some vocal fold mass lesions might block the closure of the membranous part of the vocal folds, resulting in persistent gaps and high glottal area waveform derived open quotients, which cause increased transglottic air flow, even during the most closed phase. On the one hand, this increases noise, and on the other hand, decreases the intensity of the voice source overtones due to the less abrupt interruption of the airflow [5–7]. Although vocal fold mass lesions might frequently cause dysphonia [8], not all mass lesions are necessarily associated with voice disorders. Some entities, such as swellings on the free edge of the vocal fold – frequently categorised as nodes – might develop as a consequence of vocal overuse, but do not necessarily result in dysphonic voice [9]. Neither do such swellings necessarily influence vocal fold oscillation patterns nor voice source production and are sometimes denoted as “functional” [9]. Such swellings have been observed in many professional singers without any impairment of vocal function [10, 11]. Thus, as far as there is no suspicion that these swellings are malignant, any indication for surgery should be based on functional aspects rather than on the visual mass lesion itself.

The impairment of vocal function stemming from mass lesions is sometimes not easy to detect because the voice – apart from any evaluation of rough or breathy vocal quality – can be evaluated using a number of different dimensions of vocal capacity [12, 13]. Besides vocal loading capacity, the dimensions of fundamental frequency (*ƒ*_o_) range and dynamic range have been considered important and are established elements of the voice range profile [14]. Concerning the *ƒ*_o_ range, voice production should not be considered as an homogenous entity. At some points in the *ƒ*_o_ range, biomechanical properties change abruptly leading to changes in vocal quality [15, 16]. Such circumstances can contribute to the definition of vocal registers [17]. Registration events usually occur, according to different biodynamics, in critical regions. Therefore, vocal fold mass lesions frequently impair voice production to a larger extent than the usual speaking voice *ƒ*_o_ range, i.e. the modal or chest register [18].

Because of the changes in vocal fold stiffness and mass, it can be speculated that oscillation patterns would change, not only with regard to the *ƒ*_o_ range, but also under different loudness conditions. In this context, it has been shown that the phonation threshold pressure increased in patients with vocal fold mass lesions and decreased after phonomicrosurgery [19, 20]. However, greater loudness could itself have an effect on vocal fold oscillation patterns. For healthy voices, increasing loudness is associated with greater maximum flow declination rate [7], which depends on the maximum glottal area declination rate and skewing of the glottal area function [21]. It could be assumed that longer duration of collision results in better entrainment of the oscillating systems leading to stabilization of the voice source. However, such stabilization does not appear only in healthy voices. It has been shown by Brockmann-Bauser et al. that jitter values decreased with increasing loudness in patients with vocal fold mass lesions [22]. The influence of different loudness conditions on vocal fold oscillation patterns in patients with vocal fold mass lesions has, however, not yet been clarified.

This study aims to analyze the effect of gradual changes in vocal loudness on vocal fold oscillation patterns. Consistent with the quoted studies, it was hypothesized that (1) open quotient would decrease and (2) perturbation values of the glottal area waveform would decrease with increasing sound pressure level. Furthermore, due to the blockage resulting from vocal fold mass lesions it was hypothesized that (3) the agreement of the glottal area waveform derived open quotient with the electroglottographical open quotient would not be as high as in physiologically normal voices.

## Material and methods

**Fig. 1:**
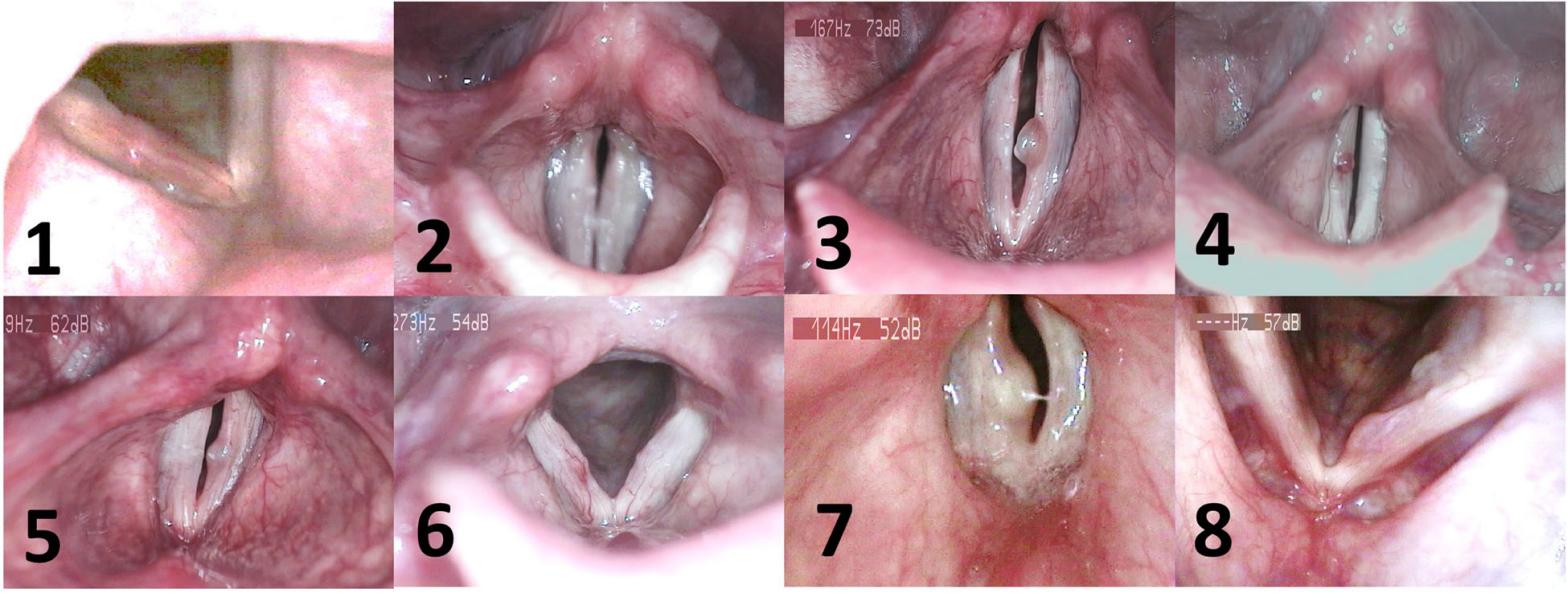
Laryngoscopic images of all subjects

After approval from the local ethical committee (Medical Ethics Committee of the University of Munich, 18/769), eight adult patients were included in the study. In order to achieve the greatest contrast of the two vocal folds, patients with unilateral predominant vocal fold mass lesions were involved. Only mass lesions were included in which an extension to the epithelium and superficial lamina propria was expected. Non-surgical therapy (i.e. voice therapy and/or pharmacotherapy) was considered not helpful for all these patients, after multidimensional voice evaluation was undertaken by an experienced phoniatrician, and consequently, phonomicrosurgery was recommended. This criterion was chosen because, one the one hand, it indicates that the mass lesion was accompanied by a dysphony and, on the other hand, could offer data if a non-surgical therapy could – in contrast to the expectation given by the decision for surgery – be meaningful. Table 1 shows age, gender, pathology, Voice Handicap Index (VHI) in the German translation [24] and the Dysphonia Severity Index (DSI) [23]. Fig. 1 displays laryngoscopic images for each subject.

**Table 1:** Gender, Age, Pathology, Lateralization, Dysphonia Severity Index (DSI) [23], Voice Handicap Index (VHI [24]) and dynamic range (from Voice Range Profile, Lingwaves, Wevosys, Forchheim, Germany)

Subject	Gender	Age (y)	Pathology	Lateralisation	DSI	VHI	Dynamical Range (dB(A))
1	f	51	edema	right	2.1	36	39
2	f	23	node	left	4.4	66	61
3	m	30	polyp	left	−1.2	25	33
4	f	60	polyp	right	3.5	12	43
5	m	28	cyst	left	4.5	23	53
6	f	43	cyst	right	2.4	48	33
7	m	38	cyst	right	5.4	33	43
8	f	59	polyp	left	−1.2	66	20

The subjects were asked to perform, on the vowel /i/, with a *ƒ*_o_ of approximately 250 Hz for the female and 125 Hz for the male voices, an increase of vocal loudness from softest to loudest. During phonation the subjects were simultaneously recorded with transnasal high speed videoendoscopy (HSV), electroglottography and audio recording.

In a similar manner to previous investigations [25, 26] high-speed videoendoscopy (HSV) (Fastcam SA-X2; Photron, Tokyo, Japan) was performed using transnasal endoscopy using a flexible endoscope (ENF GP; Fa. Olympus, Hamburg, Germany) with a frame rate of 20,000 frames per second and a spatial resolution of 386 × 320 pixels. Simultaneous to the HSV recording, the audio signal was recorded using a IMK SC 4061 microphone (DPA microphones, Alleroed, Denmark) or Sennheiser ME 62 microphone (Sennheiser, Wedemark, Germany) and electroglottographic (EGG) signals (EG2-PCX2; Glottal Enterprises, Syracuse, NY) were captured. No anesthetic medication was applied for the transnasal endoscopic approach. The audio recording was calibrated with a sound level meter (Voltcraft, Hong Kong, China) using the Sopran software (Svante Granqvist, Karolinska, Stockholm, Sweden). The HSV videos were post-processed by means of rotation, Fast-Fourier-Treatment in order to remove the comb structure of the endoscope, and cropping as previously [25]described. Calculations of the glottal area waveform (GAW) and phonovibrograms from the HSV films were performed as previously described [27, 28].

For comparison, the signals were rasterized into 100 ms time windows. Mean values for glottal area derived open quotient (OQ_GAW_), electroglottographical open quotient (OQ_EGG_), sound pressure level (SPL), Closing Quotient (Closing Phase/Period, CiQ), Speed Quotient (Opening phase/Closing phase, SQ), and fundamental frequency (*ƒ*_o_) were calculated for each window using Multi Signal Analyzer (Schäfer/Schlegel, FAU Erlangen-Nürnberg, Germany), as shown in Table 2.

**Table 2:** Measures and origin

GAW	EGG	Audio
Jitter %	Jitter %	Jitter %
		HNR
Open Quotient	Open Quotient (Howard)	
Closing Quotient		
Speed Quotient		
	Sample Entropy	
	*ƒ*_o_	
		SPL

*HNR* Harmonic-to-Noise-Ratio, *SPL* Sound pressure level, *ƒ*_o_ fundamental frequency

In order to detect OQ_GAW_ a tolerance threshold of 5% was set, i.e. that the glottis was denoted as open when the GAW signal exceeded 5% from the baseline. The electroglottographic open quotient was calculated according to the Howard criterion [29]. With regards to frequency perturbation, Jitter for all three voice signals (GAW, EGG, and audio) and the Harmonic-to-Noise-Ratio (HNR) from the audio signal were measured.

In order to compare values for a lower and greater SPL for all subjects the same difference in SPL was identified for all subjects in the following way: The minimal SPL increase during the experiment was found in subject 2, with an increase of 6 dB. Therefore, for all subjects the 100 ms window with greatest SPL and the 100 ms window with greatest SPL minus approximately 6 dB (SPL_max-6_) were compared.

The aperiodicity of vocal fold oscillation was found in many subjects at a window in between the minimum (SPL_min_) and maximum SPL (SPL_max_), and therefore the electroglottographical (EGG) sample entropy [30, 31] was used to detect the greatest changes in the EGG signals. In this respect, the window exhibiting the greatest sample entropy was denoted window 0. The 100 ms windows − 2, − 1, 0, + 1, + 2 relative to the window 0 were analysed.

The Pearson correlation test was used, but due to the small sample size comparative statistics were not considered meaningful.

## Results

**Fig. 2:**
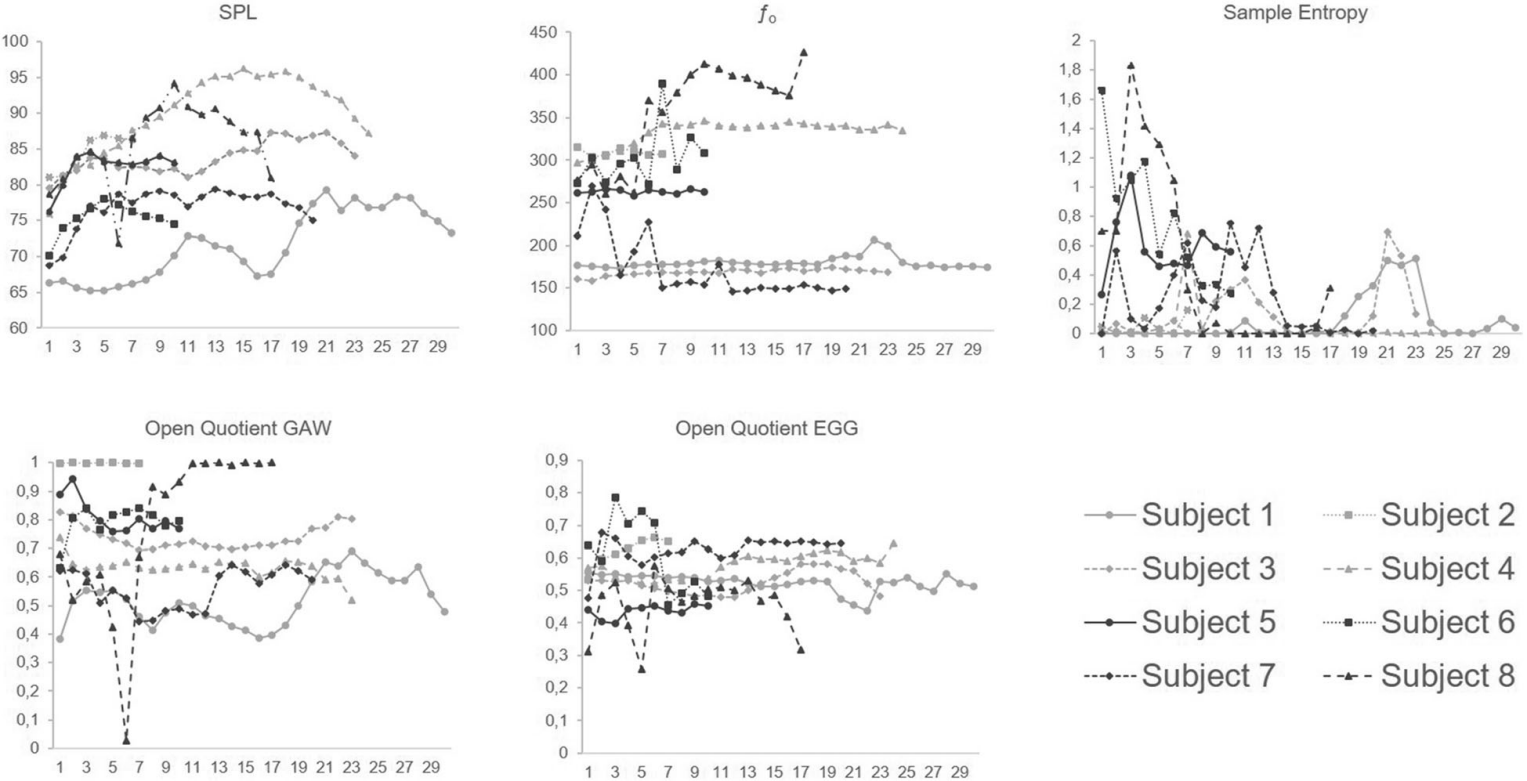
Sound Pressure Level (SPL), fundamental frequency (*ƒ*_o_), Sample Entropy, Glottal Area (GAW) and electroglottographical (EGG) derived open quotient for each 100 ms time window. The numbers on the x axis refer to each 100 ms window over the course of the experiment

All subjects were able to perform the task with the different loudness conditions. However, the increase of SPL differed among the subjects. The difference between SPL_min_ and SPL_max_ varied from 6 dB (subject 2) to 22 dB (subject 8). Figure 2 shows the trace of SPL, *ƒ*_o_, OQ_GAW_, OQ_EGG_ and the sample entropy for all subjects over the time of the experiment recording. In subject 8 for the 100 ms window 6 there was a drop of OQ_GAW_ to zero which was caused by a near total ventricular fold adduction. This window was excluded from later examinations of the SPL_max_ and SPL_max-6_ and the analysis of windows with regard to the greatest sample entropy.

**Fig. 3:**
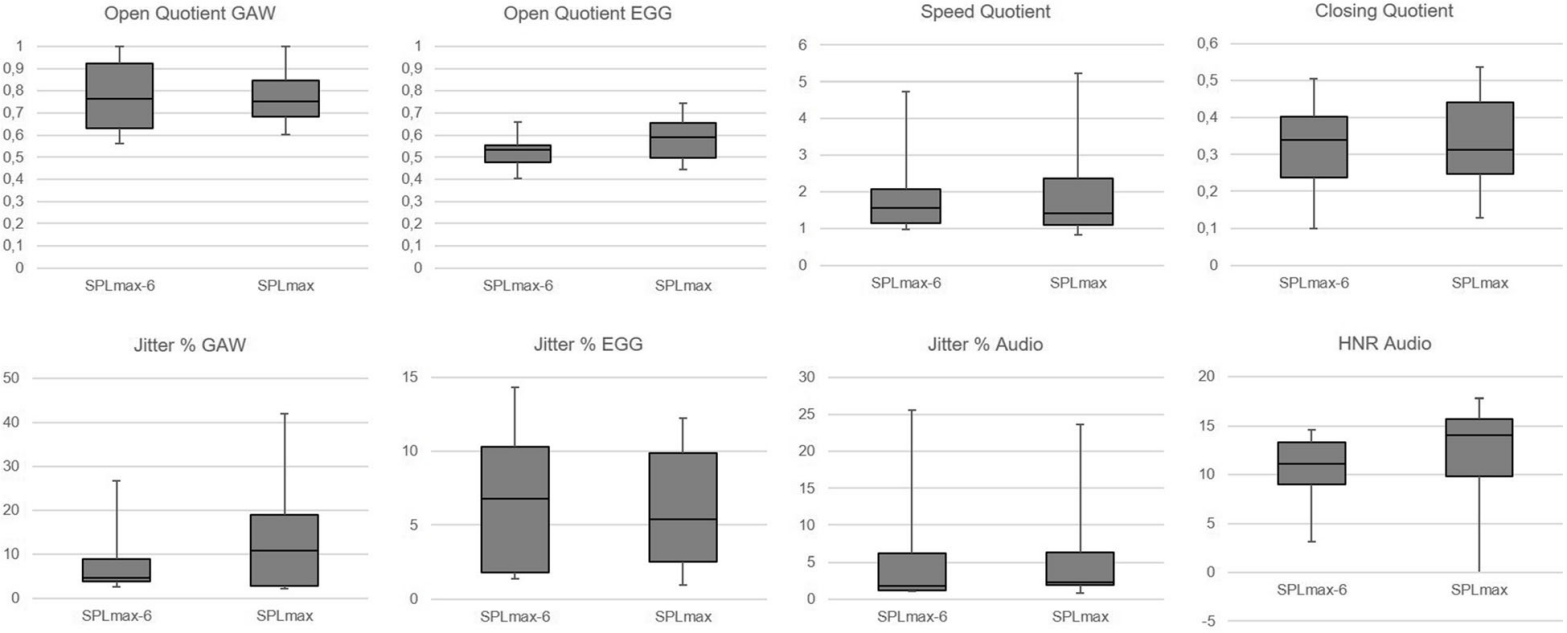
Box Plots for the window where the maximum SPL (SPL_max_, right columns) and where the maximum minus 6 dB where measured (SPL_max-6_) with respect to Glottal Area Waveform (GAW) and electroglottographical (EGG) open quotient, speed quotient, closing quotient, GAW, EGG and audio derived jitter and Harmonic to Noise Ratio (HNR)

**Fig. 4:**
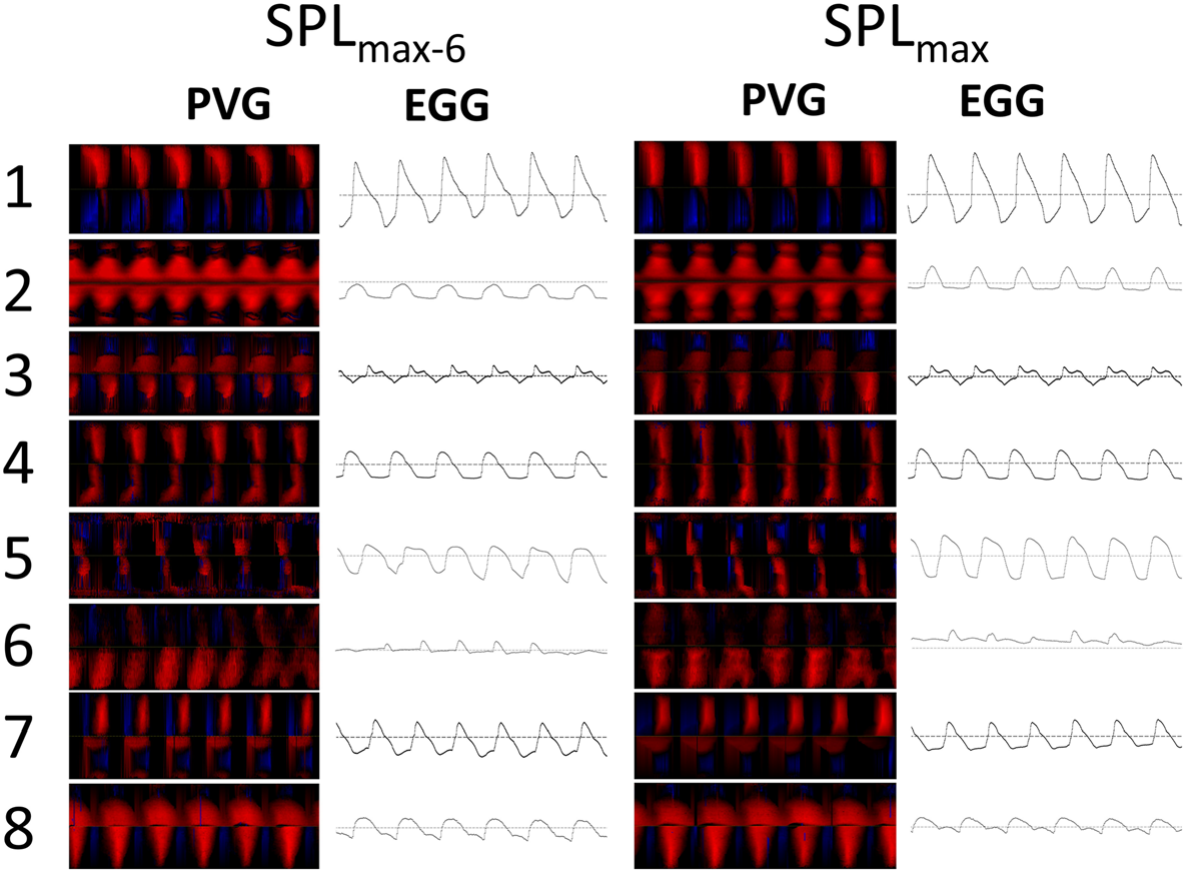
Phonovibrograms (PVGs) and electroglottographical (EGG) signals of all subjects for a 25 ms window for SPL_max-6_ (left) and SPL_max_ (right)

For the 100 ms window exhibiting SPL_max_, GAW related measures (OQ_GAW_, SQ, CiQ) showed no large difference to SPL_max-6,_ Fig. 3; in contrast, OQ_EGG_ was greater for SPL_max_. Jitter_GAW_ showed greater values for SPL_max_ whereas Jitter_Audio_ and Jitter_EGG_ showed no large difference to SPL_max-6_. The HNR was higher for SPL_max_ in comparison to SPL_max-6_. Figure 4 represents phonovibrograms for a 25 ms time interval at the mid-point of the 100 ms windows for SPL_max_ and SPL_max-6_, respectively.

**Fig. 5:**
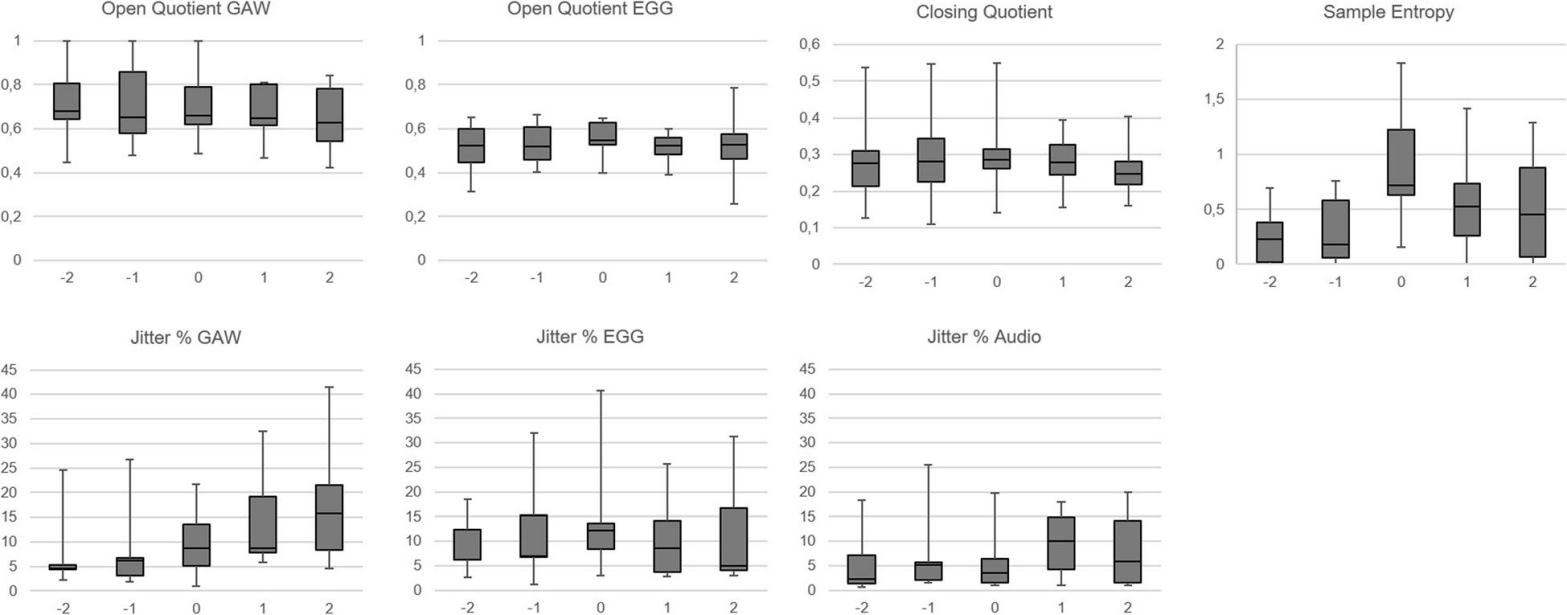
Open Quotients for GAW and EGG, Closing Quotient, Sample Entropy and Jitter for GAW, EGG and audio for the − 2 to + 2100 ms windows with respect to the window in which the greatest sample entropy was measured (0 window)

The expected *ƒ*_o_, i.e. 125 Hz for male and 250 Hz for female voices, was not achieved by many of the subjects. Some subjects (subjects 4, 6 and 8 (increased *ƒ*_o_ during the experiment), subject 7 (decreased *ƒ*_o_ during the experiment)) showed greater deviations from the required *ƒ*_o._ (Fig. 2). During the experiment, the greatest vocal instability was found between SPL_max_ and SPL_min_ for all but one subject. In the windows where the greatest sample entropy occurred, irregularities of the EGG signal and an increase in OQ_EGG_ were also found (Fig. 5). However, in the same windows, there were no large changes in the GAW; in addition neither OQ_GAW_ nor the Closing Quotient showed large changes in the 0 window in which the EGG based greatest sample entropy occurred.

**Fig. 6:**
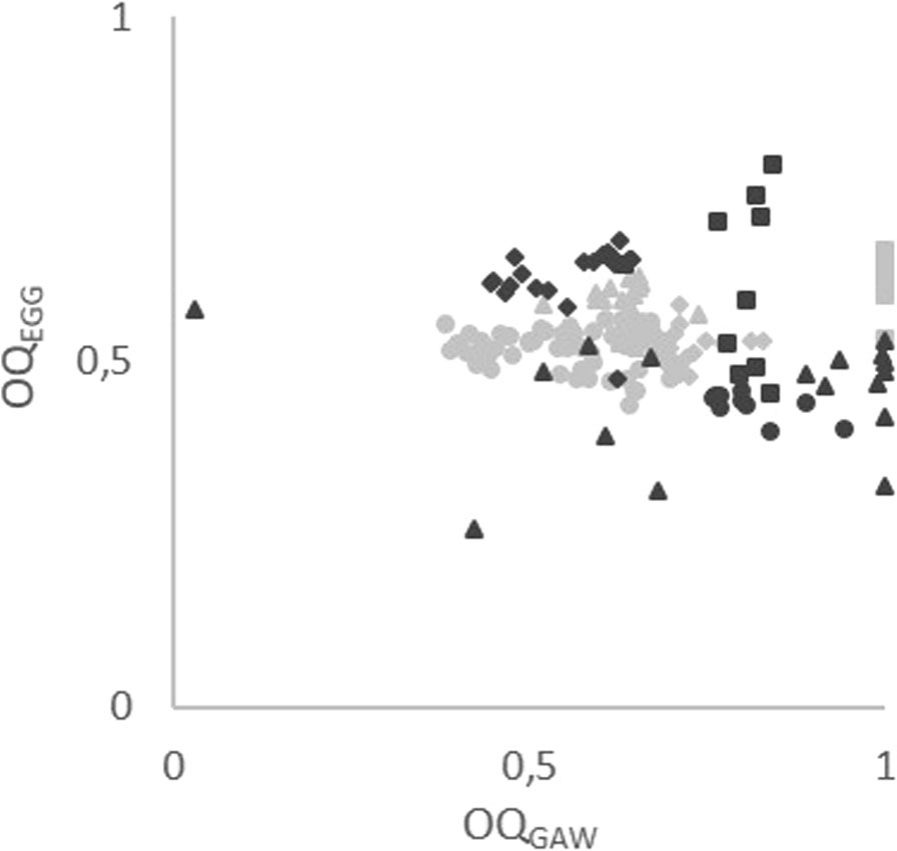
Open Quotients for GAW versus EGG

There was no correlation (trend-line equation: y = − 0,0393x + 0,5643, r = 0,084) for OQ_GAW_ and OQ_EGG,_ Fig. 6.

## Discussion

This study analyzed the effect of gradual loudness changes on vocal fold oscillation patterns. In general, for most subjects, the greatest irregularity was not found at the lowest SPL, but in between the minimum and maximum SPL. Consequently, the data presented here were not able to support the general assumption that the voice is generally stabilized with increasing SPL. Finally, there were indeed strong differences between GAW derived and EGG derived measures.

Vocal performance depends heavily on both frequency and dynamic range [1, 14]. These vocal dimensions are not only important for non-dysphonic voices but also for subjects with vocal impairments arising from vocal fold mass lesions. It has previously been shown that *ƒ*_o._ might affect vocal performance in professional singer subjects with vocal mass lesions [18]. In contrast to the previous study, no professional singers were examined in the present study and this could be considered the main reason why the required *ƒ*_o_ was frequently not achieved. However, the increase in loudness was found to be accompanied by an increase in SPL for all of the subjects. It should be noticed, however, that the subjects failed to reach the same dynamic range as they did during the clinical testing of the voice range profile. There are many potential reasons for this. One is that the time of the experiment was limited to a recording time of 9 s, producing 32 GB of HSV data, whereas during the voice range profile it was possible to make many repetitions. Another reason is that the transnasal laryngoscope might have influenced voice production arising from increased tension.

The present study hypothesized that regularity of vocal fold oscillations would increase with increasing loudness. In this respect, Brockmann-Bauser et al. [22] observed lower perturbation values derived from audio signals for higher SPL in patients with vocal fold mass lesions as well as in subjects without dysphonia. The data presented here, however, failed to support these findings: The jitter_Audio_ and jitter_EGG_ were almost unchanged between SPL_max_ and SPL_max-6_. Furthermore, for SPL_max_, jitter_GAW_ was increased. There are many possible influencing factors, which could contribute to the differences between the findings presented here and the observations made by Brockmann-Bauser et al. [22]. One is that – as noted previously –the dynamic range was lower during the experiment than in the clinical voice evaluation. Furthermore, the data presented refer to the dynamic range of 6 dB which was the lowest observed difference between the minimum and maximum SPL for subject 2. On the one hand, this provides comparability among the subjects. On the other hand, the difference of 6 dB could be considered too small to exhibit greater differences for patients who exhibited a larger dynamic range. Finally, Brockmann-Bauser et al. [22] analyzed audio signals in female voices, only. In the present study a greater number of additional signals were simultaneously analyzed which prevented a study using a larger number of subjects. Last, in the presented study two subjects (subjects 6 and 8) had a greater rise of *ƒ*_o_ during the experiment. Using sinusoidal tones, it has been shown before that a rise of *ƒ*_o_ could be associated with changes of jitter measurements [32]. At least for subject 6 this could in part explain greater jitter values for greater SPL. However, for subject 8 this tendency was present only for the jitter_Audio_ but not for the jitter_EGG_ and jitter_GAW_.

The greatest irregularities were found in between minimum and maximum SPL. With regards to changes in *ƒ*_o_ previous investigations [18] observed regions, i.e. the passaggio regions, were subjects with vocal fold mass lesions showed greater irregularity of vocal fold oscillations. In the present study, however, there were no clear criteria or regions where irregularity appeared more likely for changes in loudness and the physical value SPL.

HSV derived vocal fold oscillation patterns did not differ greatly between SPL_max_ and SPL_max-6_ with respect to OQ_GAW_, SQ and CiQ. Furthermore, as is seen in the phonovibrograms, there was no lateralization effect, i.e. the pathologic vocal fold did not behave differently to the healthy one. It is interesting that in contrast, OQ_EGG_ showed greater values for SPL_max_. It should be noted that OQ_GAW_ and OQ_EGG_ are not equivalent. OQ_GAW_ is derived from a superior laryngoscopic two-dimensional view, whereas OQ_EGG_ represents the changes in impedance due to the three-dimensional vocal fold contact. it has been shown that, in physiologic voices, the concordance of EGG and GAW signals is greater for the ‘de-contacting’ than for the ‘contacting’ phase [32]. Furthermore, for OQ_GAW_ lower than .7, the agreement of OQ_GAW_ and OQ_EGG_ is high, but for values above 0.7 this agreement is rather low [26]. The data presented here show that, for patients with vocal fold mass lesions, the disagreement for both OQs is much stronger. It could therefore be speculated that impedance changes show an earlier contact of the vocal folds due to the contact of the mass lesion, although the laryngoscopic closure still reveals open parts alongside the mass lesion. Consequently, OQ_EGG_ has to be interpreted with caution in patients with vocal fold mass lesions. Furthermore, the EGG based sample entropy was used as a criterion to describe the greatest instability in the vocal fold oscillation patterns. This measure was first introduced by Selamtzis and Ternström for analysis of physiologic voices [30]. It has been shown in non-pathologic voices that registration events can be detected using this measure [31, 33]. However, the data presented showed that the GAW derived irregularities behave differently to the EGG derived data in the time domain. Therefore, any doubts are justified as to whether the EGG based sample entropy can be used for voice evaluation in patients with vocal fold mass lesions.

There are many key limitations of this study. The first limitation stems from the variety of different mass lesion entities which are present. In this study patients with polyp, cysts, node and edema were included. Since the histopathology of the Reinke space differs specifically, the effect on stiffness and vocal fold closure could be expected to be varied. However, it should be noted that for most subjects the greatest sample entropy was not found at the limits of the dynamic range. Also in this respect, only patients with an indication for phonomicrosurgery were included. It remains unclear whether results would be comparable in patients with vocal fold mass lesions, but with a lesser impact on vocal function and, therefore, with no indication for surgery. Also in this context, the study included only patients with predominantly unilateral vocal fold mass lesions. It cannot be excluded that bilateral mass lesions would exhibit different results. As previously noted, the patients were not vocally trained and, therefore, they were not able to achieve the *ƒ*_o_ required in each case. Rising *ƒ*_o_ is frequently associated with greater SPL [7, 17]. Therefore, for subjects exhibiting greater *ƒ*_o_ changes throughout the experiment, part of the differences observed could be related not only to SPL but also to differences in *ƒ*_o_. Different loudness conditions frequently show different vocal tract shapes [34]; as such vocal tract/voice source interactions [35–37] could have influenced the observed vocal fold irregularities in different ways. Also in this respect, SPL_max_ and SPL_max-6_ were used in to compare differences for the various measures. The reason to not use the minimal SPL was that the minimum SPL was frequently found in the voice onset, and that could have a greater impact on the GAW related measures. Furthermore, the signal to noise ratio is lower for lower SPL. However, it cannot be ignored that softer loudness might exhibit a different sensitivity to the measures used.

A further important limitation is that the increase in loudness was not standardized, i.e. the increase in loudness had to be performed over a specific time interval. It could be assumed that coordination and stabilization of the voice might be easier over a longer duration, and therefore would exhibit smaller irregularity. How much the different durations in such experiments influence any irregularity should be analyzed in future investigations. Furthermore, due to the extended recording and analysis setup only eight subjects could be included in this study, which prevented any statistical analysis. It is hoped that greater numbers of subjects can be included in future investigations in order to statistically verify any observed tendencies.

## Conclusions

The amount of vocal fold irregularity changes with varying loudness. Therefore, an evaluation of voice under different loudness conditions should be recommended in patients with vocal fold mass lesions. With respect to perturbation values, this study failed to verify lower jitter values for greater SPL. The measures from electroglottographic signals and glottal area waveform differed – and therefore OQ – to a larger extent in patients with vocal fold mass lesions compared to physiologic voices.

## Data Availability

All data are available on request to the first author Matthias Echternach, Division of Phoniatrics and Pediatric Audiology, University of Munich, Munich, Germnay.

## Abbreviations

CiQ: Closing Quotient
*ƒ*_o_: fundamental frequency
HNR: Harmonic-to-Noise-Ratio
HSV: High-speed videoendoscopy
OQ_GAW_: Glottal area derived open quotient
OQ_EGG_: Electroglottographical open quotient
SPL: Sound pressure level
SQ: Speed Quotient

## References

[1] Sataloff RT (2017). Professional voice: the science and art of clinical care.

[2] Zhang Y, Jiang JJ (2008). Asymmetric spatiotemporal chaos induced by a polypoid mass in the excised larynx. Chaos.

[3] Döllinger M, Rosanowski F, Eysholdt U, Lohscheller J (2008). Basic research on vocal fold dynamics: three-dimensional vibration analysis of human and canine larynges. HNO.

[4] Oren L, Khosla S, Gutmark E (2016). Effect of vocal fold asymmetries on glottal flow. Laryngoscope.

[5] Döllinger M, Kniesburges S, Berry DA (2018). Investigation of phonatory characteristics using ex vivo rabbit larynges. J Acoust Soc Am.

[6] Birk V, Kniesburges S, Semmler M (2017). Influence of glottal closure on the phonatory process in ex vivo porcine larynges. J Acoust Soc Am.

[7] Sundberg J. The science of the singing voice. Northern Illinois University Press, 1987.

[8] Powell ME, Deliyski DD, Zeitels SM et al. Efficacy of Videostroboscopy and High-Speed Videoendoscopy to Obtain Functional Outcomes From Perioperative Ratings in Patients With Vocal Fold Mass Lesions. J Voice Epub ahead of print April 17^th^, 2019. 10.1016/j.jvoice.2019.03.012 PMC6801021 31005449

[9] Seidner W, Wendler J (2004). Die Sängerstimme.

[10] Echternach M, Burk F, Rose F (2018). Impact of functional mass lesions in professional female singers : biomechanics of vocal fold oscillation in the register transition regions. HNO.

[11] Echternach M, Arndt S, Zander M, Richter B (2009). Stimmdiagnostik bei professionellen Sängerinnen - Anwendung des Protokolls der Europäischen Laryngologischen Gesellschaft (ELS). HNO.

[12] Patel RR, Awan SN, Barkmeier-Kraemer Jet al. Recommended protocols for instrumental assessment of voice: American speech-language-hearing association expert panel to develop a protocol for instrumental assessment of vocal function. Am J Speech Lang Pathol 2018; 27:887–905. 10.1044/2018_AJSLP-17-0009 29955816

[13] Dejonckere PH, Bradley P, Clemente P (2001). A basic protocol for functional assessment of voice pathology, especially for investigating the efficacy of (phonosurgical) treatments and evaluating new assessment techniques. Guideline elaborated by the committee on Phoniatrics of the European laryngological society (ELS). Eur Arch Otorhinolaryngol.

[14] Baken RJ, Orlikoff RF (2000). Clinical measurement of speech and voice.

[15] Henrich N (2006). Mirroring the voice from Garcia to the present day: some insights into singing voice registers. Logoped Phoniatr Vocol.

[16] Echternach M, Burk F, Koberlein M (2017). Laryngeal evidence for the first and second passaggio in professionally trained sopranos. PloS One.

[17] Titze IR (1994). Principles of voice production.

[18] Echternach M, Burk F, Burdumy M. et al. The influence of vocal fold mass lesions on the passaggio region of professional singers. Laryngoscope 2017; 127:1392–1401. 10.1002/lary.26332 27753103

[19] Zhuang P, Swinarska JT, Robieux CF, Hoffman MR, Lin S, Jiang JJ (2013). Measurement of phonation threshold power in normal and disordered voice production. Ann Otol Rhinol Laryngol.

[20] Wang TG, Shau YW, Hsiao TY (2010). Effects of surgery on the phonation threshold pressure in patients with vocal fold polyps. J Formos Med Assoc.

[21] Oren L, Khosla S, Gutmark E. Medial Surface Dynamics as a Function of Subglottal Pressure in a Canine Larynx Model. J Voice Epub ahead of print August 3^rd^, 2019. 10.1016/j.jvoice.2019.07.015 PMC6995767 31387765

[22] Brockmann-Bauser M, Bohlender JE, Mehta DD (2018). Acoustic perturbation measures improve with increasing vocal intensity in individuals with and without voice disorders. J Voice.

[23] Wuyts FL, De Bodt MS, Molenberghs Get al. The dysphonia severity index: an objective measure of vocal quality based on a multiparameter approach. J Speech Lang Hear Res 2000; 43:796–809. 10.1044/jslhr.4303.796 10877446

[24] Nawka T, Wiesmann U, Gonnermann U (2003). Validation of the German version of the voice handicap index. HNO.

[25] Echternach M, Burk F, Koberlein M. et al. Oscillatory Characteristics of the Vocal Folds Across the Tenor Passaggio. J Voice 2017; 31:381 e385–381 e314. 10.1016/j.jvoice.2016.06.015 27499033

[26] Echternach M, Burk F, Koberlein M, Burdumy M, Dollinger M, Richter B (2017). The influence of vowels on vocal fold dynamics in the Tenor's Passaggio. J Voice.

[27] Lohscheller J, Eysholdt U (2008). Phonovibrogram visualization of entire vocal fold dynamics. Laryngoscope.

[28] Lohscheller J, Eysholdt U, Toy H, Dollinger M (2008). Phonovibrography: mapping high-speed movies of vocal fold vibrations into 2-D diagrams for visualizing and analyzing the underlying laryngeal dynamics. IEEE Trans Med Imaging.

[29] Howard DM (1995). Variation of electrolaryngographically derived closed quotient for trained and untrained adult female singers. J Voice.

[30] Selamtzis A, Ternstrom S (2014). Analysis of vibratory states in phonation using spectral features of the electroglottographic signal. J Acoust Soc Am.

[31] Selamtzis A, Ternstrom S, Richter B, Burk F, Koberlein M, Echternach M (2018). A comparison of electroglottographic and glottal area waveforms for phonation type differentiation in male professional singers. J Acoust Soc Am.

[32] Echternach M, Sundberg J, Zander MF, Richter B (2011). Perturbation measurements in untrained male voices' transitions from modal to falsetto register. J Voice.

[33] Wade L, Hanna N, Smith J, Wolfe J (2017). The role of vocal tract and subglottal resonances in producing vocal instabilities. J Acoust Soc Am.

[34] Echternach M, Burk F, Burdumy M, Traser L, Richter B (2016). Morphometric differences of vocal tract articulators in different loudness conditions in singing. PLoS One.

[35] Titze IR (2008). Nonlinear source-filter coupling in phonation: theory. J Acoust Soc Am.

[36] Titze IR, Riede T, Popolo P (2008). Nonlinear source-filter coupling in phonation: vocal exercises. J Acoust Soc Am.

[37] Zanartu M, Mehta DD, Ho JC, Wodicka GR, Hillman RE (2011). Observation and analysis of in vivo vocal fold tissue instabilities produced by nonlinear source-filter coupling: a case study. J Acoust Soc Am.

